# Supplementary material for: Domain-invariant features for mechanism of action prediction in a multi-cell-line drug screen
Source: Bioinformatics. 2019 Oct 14;36(5):1607–13. doi: 10.1093/bioinformatics/btz774 (PMC7058179; doi:10.1093/bioinformatics/btz774)
Supplement: btz774_Supplementary_Data [file btz774_supplementary_data.zip › btz774-Suppl_Data/SFigure2.pdf]

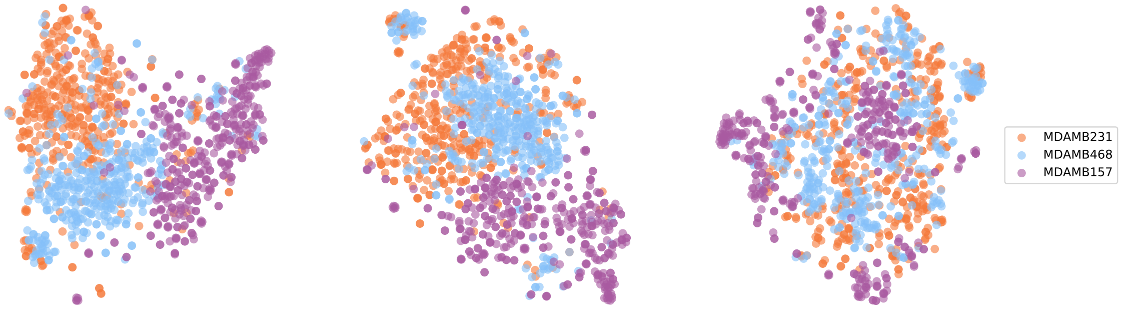

Figure 1. t-SNE embeddings of encodings from handcrafted features (left), autoencoder (center) and domain-adversarial autoencoder (right), with cell lines distinguished by colour. Respective silhouette scores of 0.22 and 0.14 and -0.02 confirm the reduced divergence in the adapted domains.
