# Supplementary material for: Domain-invariant features for mechanism of action prediction in a multi-cell-line drug screen
Source: Bioinformatics. 2019 Oct 14;36(5):1607–13. doi: 10.1093/bioinformatics/btz774 (PMC7058179; doi:10.1093/bioinformatics/btz774)
Supplement: btz774_Supplementary_Data [file btz774_supplementary_data.zip › btz774-Suppl_Data/SFigure1.pdf]

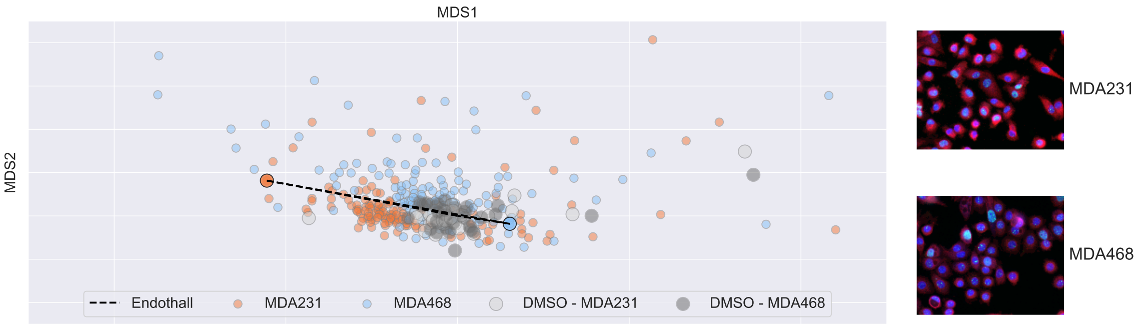

(A) Endothall takes effect in cell line MDA231 only.

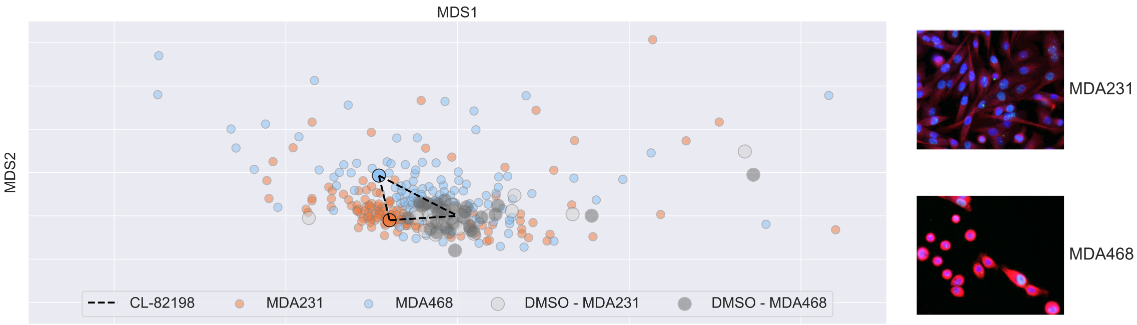

(B) CL-82198 takes effect in cell line MDA468 only.

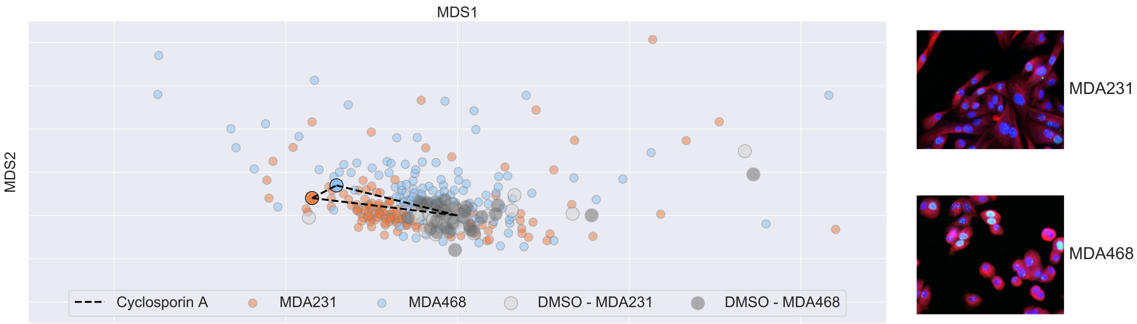

(C) Cyclosporin A takes a similar in both cell lines.

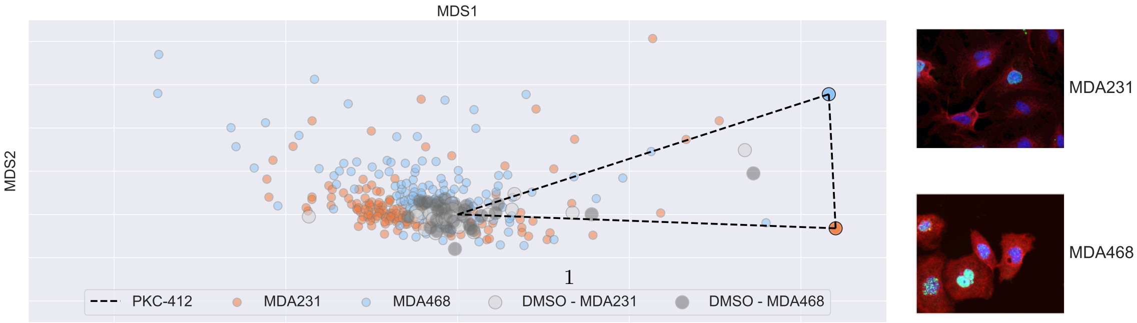

(D) PKC-412 takes differential effects in the two cell lines.

Figure 1. MDS plots of each category of drug effect. The distances between the profiles are plotted as a line, as well as the respective distances to the centroid (origin).
